# Association between *GLO1* variants and gestational diabetes mellitus susceptibility in a Chinese population: a preliminary study

**DOI:** 10.3389/fendo.2023.1235581

**Published:** 2023-11-03

**Authors:** Qiaoli Zeng, Taili Yang, Wenfeng Wei, Dehua Zou, Yue Wei, Fengqiong Han, Jieyun He, Jinzhi Huang, Runmin Guo

**Affiliations:** ^1^ Department of Internal Medicine, Shunde Women and Children’s Hospital (Maternity and Child Healthcare Hospital of Shunde Foshan), Guangdong Medical University, Foshan, Guangdong, China; ^2^ Key Laboratory of Research in Maternal and Child Medicine and Birth Defects, Guangdong Medical University, Foshan, Guangdong, China; ^3^ Maternal and Child Research Institute, Shunde Women and Children’s Hospital (Maternity and Child Healthcare Hospital of Shunde Foshan), Guangdong Medical University, Foshan, Guangdong, China; ^4^ State Key Laboratory of Quality Research in Chinese Medicine, School of Pharmacy, Macau University of Science and Technology, Taipa, Macao, Macao SAR, China; ^5^ Guangdong Engineering Research Center of Chinese Medicine & Disease Susceptibility, Jinan University, Guangzhou, Guangdong, China; ^6^ Department of Ultrasound, Shunde Women and Children’s Hospital (Maternity and Child Healthcare Hospital of Shunde Foshan), Guangdong Medical University, Foshan, Guangdong, China; ^7^ Department of Ultrasound, Affiliated Hospital of Guangdong Medical University, Zhanjiang, Guangdong, China; ^8^ Department of Obstetric, Shunde Women and Children’s Hospital (Maternity and Child Healthcare Hospital of Shunde Foshan), Guangdong Medical University, Foshan, Guangdong, China; ^9^ Department of Gynecology, Shunde Women and Children’s Hospital (Maternity and Child Healthcare Hospital of Shunde Foshan), Guangdong Medical University, Foshan, Guangdong, China; ^10^ Department of Endocrinology, Affiliated Hospital of Guangdong Medical University, Zhanjiang, Guangdong, China

**Keywords:** gestational diabetes mellitus (GDM), *GLO1*, rs1781735, rs4746, rs1130534, MG

## Abstract

**Background:**

*Glyoxalase 1* (*GLO1*) plays a crucial role in defending against glycation. Single nucleotide polymorphism (SNP) variants in the *GLO1* gene may affect gene expression and alter enzyme activity. However, there have been limited studies evaluating the association between *GLO1* and diabetes, especially gestational diabetes mellitus (GDM). Therefore, this study is the first to explore the association of *GLO1* SNPs and GDM risk.

**Methods:**

The study included a total of 500 GDM patients and 502 control subjects. The SNPscan™ genotyping assay was used to genotype rs1781735, rs4746 and rs1130534. To assess the disparities in genotype, allele, and haplotype distributions and their correlation with GDM risk, the independent sample t-test, logistic regression, and chi-square test were employed during the data processing phase. Furthermore, one-way ANOVA was conducted to determine the differences in genotype and blood glucose and methylglyoxal(MG) levels.

**Results:**

Significant differences were observed in prepregnancy body mass index (pre-BMI), age, systolic blood pressure (SBP), diastolic blood pressure (DBP), and parity between GDM and healthy subjects (*P* < 0.05). After adjusting for these factors, *GLO1* rs1130534 TA remained associated with an increased risk of GDM (TA *vs*. TT + AA: OR = 1.320; 95% CI: 1.008-1.728; *P* = 0.044), especially in the pre-BMI ≥ 24 subgroup (TA *vs*. TT + AA: OR = 2.424; 95% CI: 1.048-5.607; *P* = 0.039), with fasting glucose levels being significantly elevated in the TA genotype compared to the TT genotype (*P* < 0.05). Conversely, the *GLO1* rs4746 TG was associated with a decreased risk of GDM (TG *vs*. TT: OR = 0.740; 95% CI: 0.548-0.999; *P* = 0.049; TG *vs*. TT + GG: OR = 0.740; 95% CI: 0.548-0.998; *P* = 0.048). Additionally, the haplotype T-G-T of rs1781735, rs4746 and rs1130534 was associated with a decreased risk of GDM among individuals with a pre-BMI ≥ 24 (OR = 0.423; 95% CI: 0.188-0.955; *P* = 0.038). Furthermore, the rs1781735 GG genotype was found to be more closely related to maternal MG accumulation and neonatal weight gain (*P* < 0.05).

**Conclusion:**

Our findings suggested that *GLO1* rs1130534 was associated with an increased susceptibility to GDM and higher blood glucose levels, but *GLO1* rs4746 was associated with a decreased risk of GDM. The rs1781735 has been associated with the accumulation of maternal MG and subsequent weight gain in neonates.

## 1 Introduction

Gestational diabetes mellitus (GDM) is a common pregnancy disorder in women (1), with a prevalence ranging from 10% to 15% (2). GDM has detrimental consequences on both maternal and fetal development and increases the risk of developing type 2 diabetes mellitus (T2DM) in postpartum women (3). Previous research has proposed that the pathogenesis of GDM may be related to genetic factors (4). Therefore, single nucleotide polymorphism (SNP) variants may be associated with the development of GDM (5).

The glyoxalase 1 (*GLO1*) plays a crucial biological role in detoxifying methylglyoxal (MG) (6). Elevated levels of MG have been linked to diabetes, cardiovascular disease, and cancer (7–9), potentially due to the down-regulation of *GLO1* expression and activity (10–12). *GLO1* gene SNP variants may impact its expression and activity and have been associated with diabetes risk (13–16). Specifically, the CA genotype and C allele of *GLO1* rs4647 have been shown to increase the risk of T2DM, while the AT genotype and A allele of rs1130534 are associated with decreased susceptibility to T2DM. Notably, T2DM patients have been found to exhibit significantly increased serum MG concentrations (17). Furthermore, the minor allele of rs1130534 and rs1049346 has been related to decreased enzyme activity, with an increase in the number of risk alleles closely associated with decreased *GLO1* activity (18). These findings suggest that polymorphic variation independently impacts *GLO1* activity, with *GLO1* SNP potentially contributing to decreased enzyme activity and increased susceptibility to T2DM. Interestingly, the CC genotype of rs4647 has been associated with T2DM neuropathy (19).

The association between *GLO1* SNPs and diabetes, particularly GDM, has not been extensively researched. This study aims to investigate the association between *GLO1* rs1781735, rs4746 and rs1130534 polymorphisms and GDM and MG. The study seeks to determine the effect of *GLO1* polymorphic variants on GDM.

## 2 Materials and methods

### 2.1 Study subjects

The study enrolled 1002 participants, including 500 GDM patients and 502 healthy pregnant women as control subjects. The enrollment criteria consisted of several requirements: participants must have given written informed consent voluntarily, be Han Chinese ethnicity, aged 18 years or older, have no pregnancy complications, and not use glucose-lowering medications. The Obstetrics Clinic of Shunde Maternal and Child Health Hospital of Guangdong Medical University conducted the study between August 2021 and January 2022.

All pregnant women underwent a routine 75-gram oral glucose tolerance test (OGTT) during the 24-28 weeks of gestation. The International Association of Diabetes and Pregnancy Study Groups (IADPSG) diagnostic criteria were used to diagnose GDM. GDM was diagnosed if one or more points meet the following criteria:fasting blood glucose (FBG) ≥ 5.1 mmol/L, 1-hour postprandial glucose (PG) ≥ 10.0 mmol/L, or 2-hour PG ≥ 8.5 mmol/L. Pregnant women who did not exceed these values were included in the healthy control group.

The Ethics Committee of Shunde Maternal and Child Health Hospital of Guangdong Medical University approved the study, and it was conducted in accordance with the principles of the Declaration of Helsinki.

### 2.2 Data collection

The clinical data collected included information about ethnicity, age, pre-pregnancy weight, height, blood pressure, parity, and blood glucose levels. We calculated the pre-pregnancy body mass index (pre-BMI) by dividing the pre-pregnancy weight by the square of the height in meters. We classified obesity according to Chinese standards, which include four categories: underweight, normal, overweight, and obese.

### 2.3 SNP genotyping

Genomic DNA was extracted from whole blood using the QIAamp DNA Blood Kit from Qiagen, Germany, and then genotyped using the SNPscan method from Genesky Technologies Inc. in Shanghai, China. Quality control measures were taken to ensure the accuracy of the raw data obtained from sequencing. A subset of samples was selected for further quality control.

### 2.4 Statistical analyses

Statistical analyses were conducted using SPSS 20.0 software. Continuous variables were analyzed using independent samples t-tests for normally distributed data, and nonparametric tests were used for data that did not follow a normal distribution. The chi-square test was used for analyzing discontinuous variables, including the Hardy-Weinberg equilibrium (HWE) test for control groups. The study examined six genetic models: codominant homozygous, codominant heterozygous, dominant, recessive, overdominant, and allele models. Logistic regression was used to correct for potential confounders, and the risk of GDM was evaluated using the dominance ratio (OR) and 95% confidence interval (CI). Associations between SNP and glucose levels, neonatal weight, and MG concentrations were analyzed using one-way analysis of variance (ANOVA) with multiple comparisons (LSD) between the two groups. Subgroup analyses were conducted for age and pre-BMI. Haplotypes with a frequency below 0.03 were excluded from frequency distribution calculations. GraphPad Prism version 5.01 (GraphPad Software Inc., San Diego, CA, USA) was used to generate the statistical graphs.

### 2.5 ELISA

MG concentrations were determined by Jiangsu Meibiao Biotechnology Co. according to the manufacturer’s instructions. Standard and sample wells were prepared, with 50μL of each standard added to the standard well and 10 μL of the sample to be measured added to the sample well, followed by 40 μL of sample dilution. The blank well was left unaltered. Horseradish peroxidase (HRP)-labeled detection antibody (100μL) was added to each well, except for the blank wells. The wells were then incubated at 37°C for 60 minutes and washed five times. Subsequently, 50μL of substrate A and B were added to each well and incubated at 37°C for 15 minutes. Finally, 50μL of termination solution was added to each well, and the OD value of each well was measured at 450nm within 15 minutes.

### 2.6 Meta-analysis

A comprehensive search of the literature was conducted through the PubMed, Google Scholar, and Chinese National Knowledge Infrastructure databases for various combinations of the terms rs4746 (rs2736654), rs11305354, type 1 diabetes mellitus (T1DM), type 2 diabetes mellitus (T2DM), and gestational diabetes mellitus (GDM). Inclusion criteria included case-control or cohort studies that assessed the association of rs4746 and rs11305354 with T1DM, T2DM, or GDM, with adequate raw data. Studies that did not meet the diagnostic criteria and studies with data that were not in Hardy-Weinberg equilibrium were excluded. Two authors extracted the relevant data from the articles. Meta-analysis of six genetic models was conducted using either the fixed or random effects model, depending on the level of heterogeneity. Publication bias was assessed using Egger’s and Begg’s tests. All meta-analyses were carried out using STATA v.16.0 software.

## 3 Results

### 3.1 General clinical characteristics

We conducted a case-control study with 500 individuals diagnosed with GDM and 502 healthy controls. We examined their genotypes of *GLO1* rs1781735, rs4746 and rs1130534, and also collected basic clinical information and stratification characteristics. Our findings revealed that individuals with GDM had significantly higher mean age, pre-BMI, systolic blood pressure (SBP), diastolic blood pressure (DBP), and glucose levels compared to the control group (*P* < 0.05). Additionally, the parity (primipara/multipara) differed significantly between the two groups (*P* < 0.05). See **Table 1** for details.

**Table 1:** Basic and stratified characteristic of participants of the study.

Variables	Cases (%)	Controls (%)	t/x2	*P*
Age, year (mean ± SD)	31±4	29±4	-8.56	**< 0.001**
<30	27±2	26±3	-3.64	**< 0.001**
≥30	34±3	33±2	-3.14	**0.002**
pre-BMI, Kg/m2	21.51±3.10	20.53±2.58	-5.42	**< 0.001**
<18.5	17.45±0.84	17.60±1.50	0.75	0.453
18.5 ≤ BMI < 24	20.96±1.49	20.67±1.41	-2.63	**0.009**
≥24	26.16±2.84	25.83±3.31	-0.60	0.548
SBP, mmHg	117±11	114±10	-3.53	**< 0.001**
DBP, mmHg	70±8	68±7	-3.23	**0.001**
FBP, mmol/L	4.82±0.64	4.50±0.31	-9.75	**< 0.001**
1h-PG, mmol/L	10.17±1.60	7.66±1.27	-26.22	**< 0.001**
2h-PG, mmol/L	8.91±1.60	6.69±0.99	-25.85	**< 0.001**
Parity (n)			8.88	**0.003**
Primipara	210(42)	258(51.4)		
Multipara	290(58)	244(48.6)		

pre-BMI pre-gestational body mass index, SBP systolic blood pressure, DBP diastolic blood pressure, FBP fasting blood glucose level, 1h-PG 1 hour blood glucose level, 2h-PG 2 hour blood glucose level, bold values indicate the P < 0.05.

### 3.2 The association of rs1781735, rs4746 and rs1130534 with GDM risk

#### 3.2.1 Overall analysis results

**Table 2** presents essential details about three SNPs, including the minimal allele frequency (MAF), and the results of the Hardy-Weinberg equilibrium (HWE) analysis in the control group. A *P*-value greater than 0.05 indicates adherence to HWE. Our research results showed that the MAFs of rs1781735, rs4746, and rs1130534 are 0.355, 0.149, and 0.261, respectively. Furthermore, the control groups for each SNP are in HWE.

**Table 2:** SNPs information and HWE test in the controls.

SNP	Min/Maj	Chr. position	Region	Function	MAF	HWE (*P*)
rs1781735	G/T	chr6:38672079	5'-flanking	/	0.355	0.839
rs4746	G/T	chr6:38650628	nonsynon_exon4	p.Glu111Ala	0.149	0.431
rs1130534	A/T	chr6:38650588	synon_exon4	p.= (Gly124Gly)	0.261	0.208

HWE Hardy–Weinberg equilibrium, Min minor allele, Maj major allele, MAF frequency of minor allele.

The study evaluated the associations between six models (codominant homozygous, codominant heterozygous, dominant, recessive, overdominant and allele models) and GDM for each SNP to determine unadjusted and adjusted ORs with 95% CI and associated *P*-values. After adjusting for age, pre-BMI, SBP, DBP, and parity, *GLO1* rs1130534 showed a significant association with an increased risk of GDM in the overdominant model (TA *vs*. TT+ AA: OR = 1.320; 95% CI: 1.008-1.728; *P* = 0.044). In contrast, the heterozygous model (TG *vs*. TT. OR = 0.740; 95% CI: 0.548-0.999; *P* = 0.049) and the overdominant model (TG *vs*. TT+ GG: OR = 0.740; 95% CI: 0.548-0.998; *P* = 0.048) of *GLO1* rs4746 significantly reduced the risk of GDM. However, no significant correlation was found between *GLO1* rs1781735 and GDM (**Table 3**).

**Table 3:** The associations between *GLO1* rs1781735, rs4746 and rs1130534 and GDM risk in overall subjects.

SNP	Genetic Models	Cases (freq)	Controls (freq)	Crude OR (95 % CI)	Crude *P*	Adjusted oR (95 % CI)	Adjusted *P*
		(n=500)	(n=502)				
rs1781735	Codominant model						
	TT	205 (0.41)	210 (0.418)	1 (ref)		1 (ref)	
	TG	228 (0.456)	234 (0.466)	0.998 (0.766-1.301)	0.989	0.937 (0.708-1.239)	0.648
	GG	67 (0.134)	58 (0.115)	1.183 (0.793-1.767)	0.410	1.228 (0.805-1.872)	0.340
	Aelle model						
	T	638 (0.638)	654 (0.651)	1 (ref)		1 (ref)	
	G	362 (0.362)	350 (0.348)	1.060 (0.883-1.273)	0.531	1.055 (0.870-1.279)	0.589
	Dominant Model						
	TT	205 (0.41)	210 (0.418)	1 (ref)		1 (ref)	
	GG+TG	295 (0.59)	292 (0.582)	1.035 (0.805-1.331)	0.789	0.993 (0.762-1.294)	0.958
	Recessive Model						
	TG+TT	433 (0.866)	444 (0.885)	1 (ref)		1 (ref)	
	GG	67 (0.134)	58 (0.115)	1.185 (0.814-1.725)	0.377	1.270 (0.855-1.887)	0.236
	Overdominant model						
	TT+GG	272 (0.544)	268 (0.534)	1 (ref)		1 (ref)	
	TG	228 (0.456)	234 (0.466)	0.960 (0.749-1.231)	0.748	0.894 (0.688-1.162)	0.401
rs4746	Codominant model						
	TT	372 (0.744)	350 (0.697)	1 (ref)		1 (ref)	
	TG	119 (0.238)	143 (0.284)	0.783 (0.590-1.040)	0.091	0.740 (0.548-0.999)	**0.049**
	GG	9 (0.018)	9 (0.017)	0.941 (0.369-2.398)	0.898	0.995 (0.369-2.682)	0.991
	Aelle model						
	T	863 (0.863)	843 (0.839)	1 (ref)		1 (ref)	
	G	137 (0.137)	161 (0.16)	0.831 (0.649-1.064)	0.142	0.804 (0.619-1.042)	0.100
	Dominant Model						
	TT	372 (0.744)	350 (0.697)	1 (ref)		1 (ref)	
	GG+TG	128 (0.256)	152 (0.303)	0.792 (0.601-1.045)	0.099	0.754(0.563-1.010)	0.059
	Recessive Model						
	TG+TT	491 (0.982)	493 (0.983)	1 (ref)		1 (ref)	
	GG	9 (0.018)	9 (0.017)	1.004 (0.395-2.551)	0.993	1.073 (0.399-2.884)	0.889
	Overdominant model						
	TT+GG	381 (0.762)	359 (0.716)	1 (ref)		1 (ref)	
	TG	119 (0.238)	143 (0.284)	0.784 (0.591-1.040)	0.092	0.740 (0.548-0.998)	**0.048**
rs1130534	Codominant model						
	TT	265 (0.53)	284 (0.565)	1 (ref)		1 (ref)	
	TA	204 (0.408)	177 (0.352)	1.235 (0.951-1.605)	0.114	1.289 (0.978-1.699)	0.072
	AA	31 (0.062)	41 (0.081)	0.810 (0.494-1.330)	0.406	0.814 (0.483-1.373)	0.441
	Aelle model						
	T	734 (0.734)	745 (0.742)	1 (ref)		1 (ref)	
	A	266 (0.266)	259 (0.257)	1.042 (0.854-1.272)	0.683	1.065 (0.863-1.313)	0.558
	Dominant Model						
	TT	265 (0.53)	284 (0.565)	1 (ref)		1 (ref)	
	AA+TA	235 (0.47)	218 (0.435)	1.155 (0.901-1.482)	0.256	1.198 (0.922-1.558)	0.177
	Recessive Model						
	TA+TT	469 (0.938)	461 (0.919)	1 (ref)		1 (ref)	
	AA	31 (0.062)	41 (0.081)	0.743 (0.458-1.206)	0.229	0.734 (0.441-1.222)	0.235
	Overdominant model						
	TT+AA	296 (0.592)	325 (0.648)	1 (ref)		1 (ref)	
	TA	204 (0.408)	177 (0.352)	1.265 (0.980-1.634)	0.071	1.320 (1.008-1.728)	**0.044**

Adjusted *P* value calculated by logistic regression with adjustment for age, pre-BMI, SBP, DBP and parity, bold values indicate the *P* < 0.05.

#### 3.2.2 Stratified analysis results

We conducted stratified analyses based on age and pre-BMI to investigate the association between SNPs and GDM susceptibility in six genetic models. We found that in the subgroup of women aged less than 30 years, the *GLO1* rs1130534 recessive model significantly decreased the risk of GDM (AA *vs*. TA+TT: OR = 0.369; 95% CI: 0.145-0.935; *P* = 0.036) (**Table 4**). In contrast, in the subgroup of women with a pre-pregnancy BMI of 24 or higher, the *GLO1* rs1130534 codominant heterozygous model significantly increased the risk of GDM (TA *vs*. TT+ AA: OR = 2.424; 95% CI: 1.048-5.607; *P* = 0.039). The *GLO1* rs4746 codominant homozygous model (GG *vs*. TT: OR = 0.142; 95% CI: 0.026-0.780; *P* = 0.025), allele model (G *vs*. T: OR = 0.464; 95% CI: 0.244-0.884; *P* = 0.020), and recessive model (GG *vs*. TG+ TT: OR = 0.156; 95% CI: 0.029-0.839; *P* = 0.030) significantly decreased the risk of GDM, but no significant correlation was found after correction (**Table 5**). No significant correlation with GDM was found in any other groups (**Supplementary Tables 1-3**). Our findings suggest that certain genetic variations may affect the risk of developing GDM in specific subgroups of women based on their age and pre- BMI.

**Table 4:** The associations between *GLO1* rs1781735, rs4746 and rs1130534 and GDM risk in age < 30 subjects.

SNP	Genetic Models	Case (freq)(n=192)	Controls (freq)(n=304)	Crude OR (95 % CI)	Crude *P*	Adjusted OR (95 % CI)	Adjusted *P*
rs1781735	Codominant model						
	TT	81 (0.421)	128 (0.421)	1 (ref)		1 (ref)	
	TG	83 (0.432)	135 (0.444)	0.972 (0.658-1.435)	0.885	0.988 (0.659-1.482)	0.955
	GG	28 (0.145)	41 (0.134)	1.079 (0.619-1.880)	0.788	1.084 (0.609-1.929)	0.783
	Aelle model						
	T	245 (0.638)	391 (0.643)	1 (ref)		1 (ref)	
	G	139 (0.361)	217 (0.356)	1.022 (0.783-1.334)	0.871	1.029 (0.781-1.355)	0.840
	Dominant Model						
	TT	81 (0.421)	128 (0.421)	1 (ref)		1 (ref)	
	GG+TG	111 (0.579)	176 (0.579)	0.997 (0.6917-1.437)	0.986	1.011 (0.692-1.478)	0.955
	Recessive Model						
	TG+TT	164 (0.855)	263 (0.866)	1(ref)		1(ref)	
	GG	28 (0.145)	41 (0.134)	1.095 (0.652-1.840)	0.731	1.091 (0.636-1.869)	0.752
	Overdominant model						
	TT+GG	109 (0.568)	169 (0.556)	1 (ref)		1 (ref)	
	TG	83 (0.432)	135 (0.444)	0.953 (0.662-1.372)	0.797	0.969 (0.663-1.415)	0.870
rs4746	Codominant model						
	TT	145 (0.755)	215 (0.707)	1(ref)		1(ref)	
	TG	41 (0.213)	84 (0.276)	0.724 (0.471-1.111)	0.139	0.709 (0.455-1.105)	0.128
	GG	6 (0.031)	5 (0.016)	1.779 (0.533-5.939)	0.349	1.830 (0.511-6.551)	0.353
	Aelle model						
	T	331 (0.861)	514 (0.845)	1 (ref)		1 (ref)	
	G	53 (0.138)	94 (0.154)	0.876 (0.608-1.260)	0.474	0.863 (0.592-1.258)	0.444
	Dominant Model						
	TT	145 (0.755)	215 (0.707)	1(ref)		1(ref)	
	GG+TG	47 (0.245)	89 (0.293)	0.783 (0.519-1.182)	0.244	0.768 (0.501-1.177)	0.225
	Recessive Model						
	TG+TT	186 (0.969)	299 (0.984)	1 (ref)		1 (ref)	
	GG	6 (0.031)	5 (0.016)	1.929 (0.581-6.410)	0.284	1.992 (0.559-7.094)	0.288
	Overdominant model						
	TT+GG	151 (0.787)	220 (0.724)	1 (ref)		1 (ref)	
	TG	41 (0.213)	84 (0.276)	0.711 (0.464-1.090)	0.118	0.696 (0.447-1.083)	0.109
rs1130534	Codominant model						
	TT	102 (0.531)	171 (0.562)	1 (ref)		1 (ref)	
	TA	84 (0.437)	108 (0.355)	1.304 (0.895-1.899)	0.167	1.310 (0.887-1.935)	0.175
	AA	6 (0.031)	25 (0.082)	0.402 (0.160-1.014)	0.053	0.414 (0.161-1.067)	0.068
	Aelle model						
	T	288 (0.75)	450 (0.74)	1 (ref)		1 (ref)	
	A	96 (0.25)	158 (0.259)	0.949 (0.708-1.273)	0.729	0.958 (0.706-1.299)	0.781
	Dominant Model						
	TT	102 (0.531)	171 (0.562)	1 (ref)		1 (ref)	
	AA+TA	90 (0.469)	133 (0.438)	1.134 (0.789-1.631)	0.496	1.145 (0.786-1.670)	0.480
	Recessive Model						
	TA+TT	186 (0.969)	279 (0.918)	1 (ref)		1 (ref)	
	AA	6 (0.031)	25 (0.082)	0.360 (0.145-0.894)	**0.028**	0.369 (0.145-0.935)	**0.036**
	Overdominant model						
	TT+AA	108 (0.563)	196 (0.645)	1 (ref)		1 (ref)	
	TA	84 (0.437)	108 (0.355)	1.412 (0.976-2.042)	0.067	1.417 (0.966-2.078)	0.074

Adjusted *P* value calculated by logistic regression with adjustment for age, pre-BMI, SBP, DBP and parity, bold values indicate the *P* < 0.05.

**Table 5:** The associations between *GLO1* rs1781735, rs4746 and rs1130534and GDM risk in pre-BMI ≥ 24 subjects.

SNP	Genetic Models	Cases (freq) (n=97)	Controls (freq)(n=42)	Crude OR (95 % CI)	Crude *P*	Adjusted OR (95 % CI)	Adjusted *P*
rs1781735	Codominant model						
	TT	41 (0.422)	19 (0.452)	1 (ref)		1 (ref)	
	TG	46 (0.474)	22 (0.523)	0.969 (0.460-2.040)	0.934	0.758 (0.340-1.690)	0.498
	GG	10 (0.103)	1 (0.023)	4.634 (0.553-38.855)	0.158	4.725 (0.545-40.937)	0.159
	Aelle model						
	T	128 (0.659)	60 (0.714)	1 (ref)		1 (ref)	
	G	66 (0.34)	24 (0.285)	1.289 (0.737-2.254)	0.373	1.183 (0.658-2.127)	0.575
	Dominant Model						
	TT	41 (0.422)	19 (0.452)	1 (ref)		1 (ref)	
	GG+TG	56 (0.578)	23 (0.548)	1.128 (0.544-2.339)	0.746	0.934 (0.431-2.026)	0.863
	Recessive Model						
	TG+TT	87 (0.897)	41 (0.977)	1 (ref)		1 (ref)	
	GG	10 (0.103)	1 (0.023)	4.713 (0.584-38.059)	0.146	5.429 (0.653-45.173)	0.118
	Overdominant model						
	TT+GG	51 (0.526)	20 (0.477)	1 (ref)		1 (ref)	
	TG	46 (0.474)	22 (0.523)	0.820 (0.397-1.693)	0.591	0.634 (0.290-1.386)	0.254
rs4746	Codominant model						
	TT	73 (0.752)	26 (0.619)	1 (ref)		1 (ref)	
	TG	22 (0.226)	11 (0.261)	0.712 (0.304-1.668)	0.435	0.830 (0.336-2.051)	0.687
	GG	2 (0.02)	5 (0.119)	0.142 (0.026-0.780)	**0.025**	0.186 (0.031-1.094)	0.063
	Aelle model						
	T	168 (0.865)	63 (0.75)	1 (ref)		1 (ref)	
	G	26 (0.134)	21 (0.25)	0.464 (0.244-0.884)	**0.020**	0.543 (0.276-1.072)	0.078
	Dominant Model						
	TT	73 (0.752)	26 (0.619)	1 (ref)		1 (ref)	
	GG+TG	24 (0.248)	16 (0.381)	0.534 (0.246-1.160)	0.113	0.627 (0.276-1.425)	0.265
	Recessive Model						
	TG+TT	95 (0.98)	37 (0.881)	1 (ref)		1 (ref)	
	GG	2 (0.02)	5 (0.119)	0.156 (0.029-0.839)	**0.030**	0.194 (0.033-1.129)	0.068
	Overdominant model						
	TT+GG	75 (0.774)	31 (0.739)	1 (ref)		1 (ref)	
	TG	22 (0.226)	11 (0.261)	0.827 (0.358-1.907)	0.655	0.934 (0.382-2.286)	0.881
rs1130534	Codominant model						
	TT	45 (0.463)	25 (0.595)	1 (ref)		1 (ref)	
	TA	45 (0.463)	11 (0.261)	2.273 (1.000-5.164)	0.050	2.211 (0.931-5.250)	0.072
	AA	7 (0.072)	6 (0.142)	0.648 (0.196-2.141)	0.477	0.553 (0.157-1.943)	0.355
	Aelle model						
	T	135 (0.695)	61 (0.726)	1 (ref)		1 (ref)	
	A	59 (0.304)	23 (0.273)	1.159 (0.656-2.047)	0.611	1.088 (0.601-1.973)	0.780
	Dominant Model						
	TT	45 (0.463)	25 (0.595)	1 (ref)		1 (ref)	
	AA+TA	52 (0.537)	17 (0.405)	1.699 (0.816-3.541)	0.157	1.590 (0.737-3.433)	0.237
	Recessive Model						
	TA+TT	90 (0.928)	36 (0.858)	1 (ref)		1 (ref)	
	AA	7 (0.072)	6 (0.142)	0.467 (0.147-1.484)	0.197	0.408 (0.120-1.385)	0.150
	Overdominant model						
	TT+AA	52 (0.537)	31 (0.739)	1 (ref)		1 (ref)	
	TA	45 (0.463)	11 (0.261)	2.439 (1.101-5.402)	**0.028**	2.424 (1.048-5.607)	**0.039**

Adjusted *P* value calculated by logistic regression with adjustment for age and SBP, bold values indicate the *P* < 0.05.

### 3.3 Association between haplotype and GDM risk

Linkage disequilibrium between the three SNPs was strong(D’ > 0.85), and haplotype analysis revealed that the T-G-T haplotype of rs1781735, rs4746 and rs1130534 significantly decreased the risk of GDM in individuals with pre-BMI ≥ 24 (OR = 0.423; 95% CI: 0.188-0.955; *P* = 0.038) (**Table 6**). No significant correlation between haplotypes and GDM risk was found in other groups (**Supplementary Table 4**).

**Table 6:** Haplotype analysis of the *GLO1* rs1781735, rs4746 and rs1130534and GDM risk in pre-BMI ≥ 24 subjects.

Haplotype	Cases (freq)	Control s(freq)	OR ( 95% CI )	*P*
TTT	45 (0.231)	16 (0.19)	1 (ref)	
GTT	64 (0.329)	24 (0.285)	0.948 (0.453-1.984)	0.888
TGT	25 (0.128)	21 (0.25)	0.423 (0.188-0.955)	**0.038**
TTA	58 (0.298)	23 (0.273)	0.897 (0.425-1.893)	0.775

Bold values indicate the *P* < 0.05.

### 3.4 Association between genotype and blood glucose level

In the < 30 years age subgroup, individuals with the rs1130534 AA genotype had a significantly lower 2-hour glucose level than those with the TT and TA genotypes (*P* < 0.05) (**Table 7**). In the pre-BMI≥24 subgroup, individuals with the TA genotype of rs1130534 showed a significantly higher fasting glucose level than those with the TT genotype (*P* < 0.05) (**Table 7**). No significant differences were observed between genotypes and blood glucose levels in other groups (P ≥ 0.05) (**Supplementary Table 5**).

**Table 7:** Association between polymorphisms genotype and blood glucose level and neonatal weight.

Groups	SNP	Genotype	FBG (mmol/L)	1 h-PG (mmol/L)	2 h-PG (mmol/L)	Neonatal weight (g)
age < 30	rs1781735	TT	4.630±0.617	8.487±1.976	7.429±1.551	3156.842±379.264
		TG	4.597±0.879	8.467±1.997	7.389±1.778	3176.858±332.455
		GG	4.652±0.511	8.323±1.786	7.308±1.391	3244.362±385.925
		F	0.178	0.177	0.136	1.531
		*P*	> 0.05	> 0.05	> 0.05	> 0.05
	rs4746	TT	4.641±0.767	8.477±1.937	7.402±1.639	3178.044±359.441
		TG	4.545±0.628	8.330±2.037	7.288±1.631	3188.400±370.950
		GG	4.700±0.483	9.100±1.663	8.300±1.059	3050.000±281.069
		F	0.793	0.792	1.788	0.743
		*P*	> 0.05	> 0.05	> 0.05	> 0.05
	rs1130534	TT	4.586±0.546	8.358±1.720	7.352±1.458^b^	3203.355±351.921^a^
		TA	4.661±0.928	8.686±2.247	7.576±1.854^a^	3136.354±370.521^a^
		AA	4.655±0.814	7.931±1.944	6.690±1.466^ab^	3209.677±364.257
		F	0.583	2.569	3.906	2.082
		*P*	> 0.05	> 0.05	**< 0.05 **	> 0.05
pre-BMI < 18.5	rs1781735	TT	4.569±0.688	8.465±1.896	7.471±1.674	3085.526±376.143
		TG	4.492±0.504	8.538±1.818	7.582±1.812	3064.429±300.809^a^
		GG	4.600±0.507	8.600±1.765	7.600±1.056	3256.250±313.494^a^
		F	0.368	0.046	0.085	2.116
		*P*	> 0.05	> 0.05	> 0.05	> 0.05
	rs4746	TT	4.555±0.599	8.494±1.822	7.491±1.655	3096.410±346.279
		TG	4.500±0.604	8.424±1.869	7.574±1.797	3104.878±340.126
		GG	4.500±0.577	9.750±2.062	8.250±1.258	2882.500±178.396
		F	0.126	0.955	0.407	0.787
		*P*	> 0.05	> 0.05	> 0.05	> 0.05
	rs1130534	TT	4.519±0.503	8.501±1.739	7.530±1.579	3117.470±332.029
		TA	4.533±0.566	8.497±1.899	7.712±1.742	3034.688±311.081
		AA	4.667±1.047	8.600±2.197	6.786±1.847	3209.333±478.830
		F	0.384	0.020	1.741	2.038
		*P*	> 0.05	> 0.05	> 0.05	> 0.05
pre-BMI ≥ 24	rs1781735	TT	4.930±0.593	9.684±1.983	8.158±1.730	3314.667±385.691^a^
		TG	4.905±0.615	9.790±2.248	8.339±2.056	3334.851±350.397
		GG	4.900±0.316	9.400±1.265	8.000±1.333	3576.000±451.619^a^
		F	0.031	0.162	0.222	2.132
		*P*	> 0.05	> 0.05	> 0.05	> 0.05
	rs4746	TT	4.912±0.551	9.778±1.913	8.233±1.891	3354.485±355.423^a^
		TG	4.909±0.678	9.697±2.518	8.333±1.931	3386.970±399.425^b^
		GG	5.000±0.632	8.833±1.472	7.667±0.816	2988.571±432.567^ab^
		F	0.065	0.587	0.323	3.489
		*P*	> 0.05	> 0.05	> 0.05	**< 0.05**
	rs1130534	TT	4.813±0.500^a^	9.438±1.833	8.016±1.589	3375.362±401.192
		TA	5.056±0.656^a^	10.075±2.344	8.528±2.136	3304.636±360.405
		AA	4.833±0.577	9.583±1.782	8.083±1.881	3340.000±316.070
		F	2.734	1.423	1.146	0.536
		*P*	> 0.05	> 0.05	> 0.05	> 0.05

^a,b^A p-value<0.05 indicates statistical significance.

### 3.5 Association between genotype and neonatal weight

In the < 30 years age subgroup, the TA genotype of rs1130534 was associated with a significantly lower impact on neonatal weight compared to the TT genotype (*P* < 0.05) (**Table 7**). The GG genotype of rs1781735 had a significantly higher impact on neonatal weight than the TG genotype (*P* < 0.05) (**Table 7**). The GG genotype of rs4746 had a significantly lower impact on neonatal weight than both the TT and TG genotypes (*P* < 0.05) (**Table 7**). No significant differences were observed between genotypes and neonatal weight in other groups (P ≥ 0.05) (**Supplementary Table 5**).

### 3.6 Association between genotype and MG level

**Figure 1:**
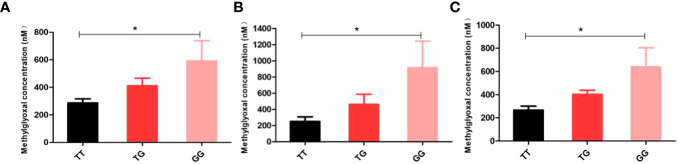
Discrimination of methylglyoxal (MG) levels in genotypes of rs1781735 in serum **(A)** in the overall subjects **(B)** in the age ≥ 30 subjects **(C)** in the 18.5 ≤ pre-BMI < 24 subjects. *P < 0.05 indicates statistical significance.

The study conducted measurements of MG levels in 34 cases and 36 controls, and analyzed the relationship between different genotypes and MG. The findings revealed that the GG genotype of rs1781735 had significantly higher levels of MG compared to the TT genotype (*P* < 0.05), particularly in the subgroups of age ≥ 30 and 18.5 ≤ pre-BMI < 24 (**Figure 1**). However, no correlation was observed between any of the genotypes and MG in the other groups (**Supplementary Table 6**).

### 3.7 Meta-analysis results

**Figure 2:**
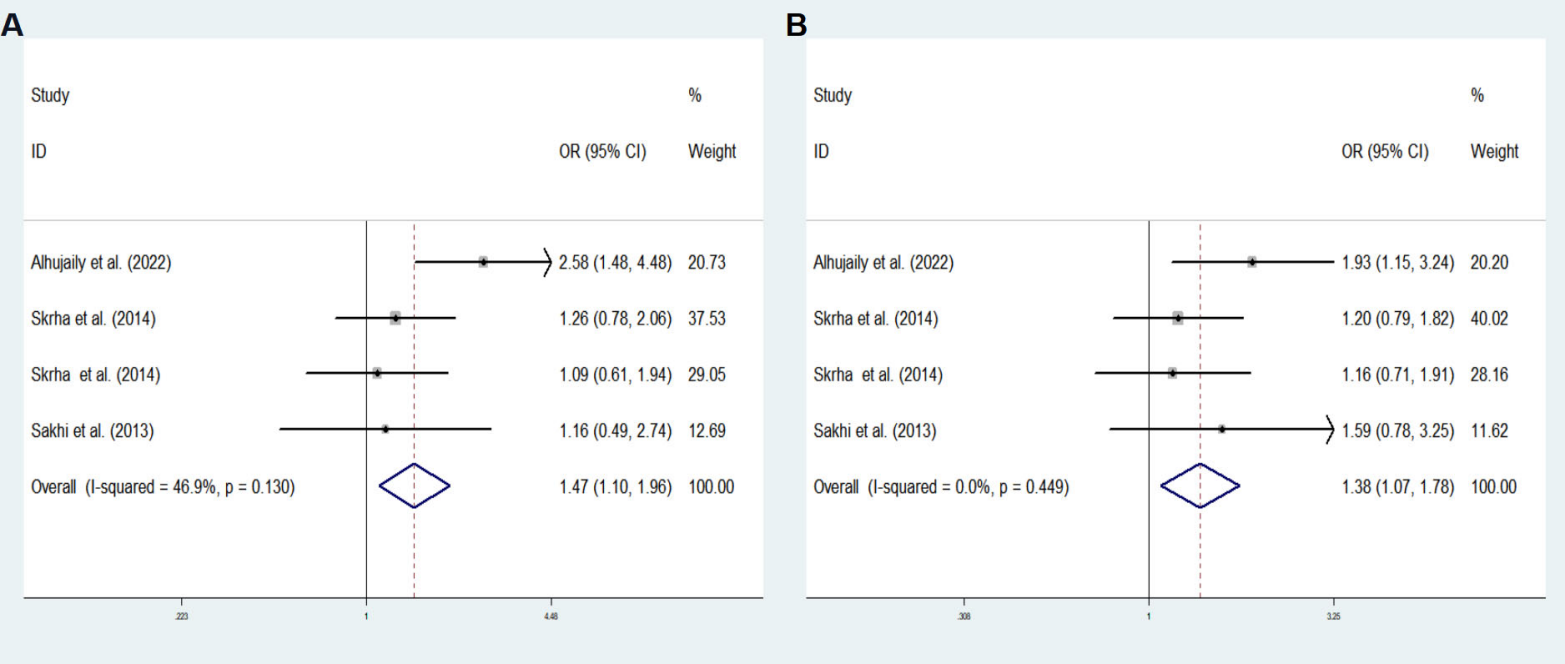
Subgroup meta-analysis for the association between *GLO1* rs4746 and DM susceptibility in a Caucasian population in fixed effects model. **(A)** Heterozygous model, TG *vs*. TT **(B)** Overdominant model, TG *vs*. TT+ GG. OR odds ratio, CI confidence interval, I^2^: measurement to quantify the degree of heterogeneity in meta-analyses.

The final analysis comprised of five studies (including our own) examining the associations of rs4746 and rs1130534 with DM (GDM, T1DM and T2DM). **Supplementary Table 7** outlines the characteristics of the studies. The overall analysis did not show any significant association between the two SNPs and DM. However, subgroup meta-analysis of the rs4746 heterozygous model (TG *vs*. TT: OR = 1.473; 95% CI: 1.105-1.964; *P* = 0.008) and the overdominant model (TG *vs*. TT+ GG: OR = 1.385; 95% CI: 1.075-1.783; *P* = 0.012) revealed a significant increase in the risk of DM (T1DM and T2DM) in the Caucasian population (**Figure 2**). No significant difference was observed in other genetic models. The results were consistent with Egger’s tests (all *P* > 0.05), suggesting no evidence of publication bias.

## 4 Discussion

In instances of diabetes and metabolic disorders, there is an increase in MG that can surpass the intracellular detoxification capacity of *GLO1*, leading to the formation of advanced glycation end products (AGEs). These AGEs may cause cellular dysfunction and tissue damage (12, 20, 21). It has been suggested that genetic variations in the *GLO1* gene may result in altered expression, conformational modifications, or enzymatic activity, resulting in an isozyme with a reduced detoxification capacity (22, 23).

One of the earliest SNPs identified in the *GLO1* gene is rs4746 (also referred to as rs2736654). This SNP is situated in the fourth exon, and the mutation rs4746 T > G results in a substitution of glutamate with alanine (24). This substitution may be linked to decreased *GLO1* enzyme activity (24). Given that glutamate is negatively charged, and alanine is uncharged at physiological pH, rs4647 A > C could potentially exert a significant influence on the structure and function of *GLO1*. According to the findings of Alhujaily et al., individuals with the rs4647 A > C genotype exhibited higher levels of fasting glucose and HbA1c. Moreover, those with the CA genotype and C allele were found to be more susceptible to developing T2DM (17). The study also revealed that patients with T2DM had significantly elevated concentrations of MG, which could be attributed to the reduced activity of the *GLO1* enzyme caused by structural perturbations. This accumulation of MG, a cytotoxic substrate, is known to cause insulin resistance and ultimately lead to the development of T2DM (25, 26).

The results of our study demonstrated that the TG genotype of rs4746 may have a protective effect against GDM. Additionally, we found that the GG genotype and G allele in the pre-BMI ≥ 24 group were associated with a lower risk of GDM before accounting for confounding factors. Interestingly, neonatal weight was found to be significantly lower in the GG genotype compared to the TG and TT genotypes. These findings align with previous research on diabetic complications and neurological disorders, where the rs4746 AA genotype was found to reduce enzyme activity (22), but not CC. It has been suggested that individuals who carry the CC genotype may have a lower incidence of diabetic neuropathy (23, 27, 28). On the other hand, the A allele is more prevalent in individuals with autism, which leads to reduced activity of the Glo1 enzyme in brain extracts and results in the accumulation of advanced AGEs in the damaged brain (22). Furthermore, lymphoblastoid cells that are homozygous for the A allele have been found to have reduced enzyme activity, resulting in elevated levels of MG and the receptor for advanced glycation end products (RAGE) (16, 24, 29). Therefore, the presence of the GLO1 A allele appears to play a role in neurological disorders associated with chronic inflammatory processes and AGE formation.

Our meta-analysis results indicated that rs4746 TG genetype was significantly increased the risk of diabetes (T1DM and T2DM) in Caucasian population, which is contrary to our results. This discrepancy could be attributed to ethnic differences. Additionally, due to the lack of studies on rs4746 and diabetes in Asian people, further research is necessary.

The *GLO1* rs1130534 T > A genetic variant results in a codon 124 synonymous substitution, which does not alter the glycine amino acid (30). Our study found that the TA genotype of rs1130534 significantly increases the risk of GDM, particularly in the pre-BMI ≥ 24 group. Correspondingly, fasting glucose levels were significantly higher in individuals with the TA genotype compared to those with the TT genotype. In individuals aged < 30 years, the AA genotype was found to be a protective factor against gestational diabetes. Furthermore, the 2-h glucose test was significantly lower in the AA genotype than in the TA and TT genotypes, and neonatal weight was significantly lower in the TA genotype compared to the TT genotype. These findings were consistent with previous research indicating that GLO-1 rs1130534 T > A and the AT genotype of the A allele are associated with reduced susceptibility to T2DM. Additionally, rs1130534 T >A was significantly different in patients with normal and elevated glucose, as well as in those with normal and abnormal lipids. The T allele of rs1130534 was found to be associated with decreased *GLO1* activity in whole blood samples, but the possible functional involvement of rs1130534 in relation to reduced *GLO1* activity remains unclear (17). However, previous research has suggested that other synonymous SNPs can alter the phenotype by disrupting gene regulation (31). Further functional studies, including the estimation of mRNA and/or protein levels, are required to confirm the function of the studied polymorphisms.

The rs1781735 variant is located in the promoter of *GLO1* and had a significant effect on the transcriptional activity of *GLO1* (32). One study in a Chinese population showed that the *GLO1* promoter containing the rs1781735 T allele had significantly lower activity than the G allele and was associated with the risk of schizophrenia (30). However, our results did not find an association between rs1781735 and GDM but seemed to be more associated with cumulative neonatal weight and MG levels. In subgroup analysis, the results showed significantly higher neonatal weight in the GG genotype than in the TG or TT genotype. The *GLO1* protection against dicarbonyl stress is crucial both developmentally and functionally (10), and further study is needed to investigate the impact of various genotypes on neonatal development. In addition, the results of MG levels measured with a limited sample size revealed that MG levels were significantly higher in the GG genotype than in the TT genotype, especially in the age ≥ 30 and 18.5 ≤ pre-BMI < 24 groups. These results suggested an influence of the GG genotype on MG accumulation. The heightened formation of MG contributes to cellular and tissue dysfunction (10), and the plasma MG level in patients with clinical diabetes was found to be elevated. Specifically, the whole blood MG concentration in patients with Type 1 Diabetes Mellitus (T1DM) increased by 5-6 times, whereas in patients with T2DM, it increased by 3-4 times, as reported in a previous study (10). However, due to limitations in sample size, we were unable to identify any significant differences in serum MG levels between patients with GDM and normal pregnant women. Nevertheless, we did observe that serum MG levels were 2-3 times higher in individuals with the GG genotype compared to those with the TT genotype, as depicted in **Figure 1**. Therefore, rs1781735 may affect mRNA expression, *GLO1* activity, and subsequently MG levels. Further functional validation of these contradictory results may be needed, and with the very limited sample size of our MG assay, further validation with an expanded sample size is essential.

Despite disregarding the limitation of sample size, our findings indicated that there was an association between rs4746 and rs1130534 with GDM, but no correlation with MG. Conversely, rs1781735 was not associated with GDM; However, it was linked to the accumulation of MG. This may be due to the insignificant clinical difference in MG caused by rs1781735. Alternatively, MG accumulation may be a factor in the pathogenesis of GDM. Therefore, we analyzed the association of rs1781735, rs4746 and rs1130534 haplotypes with gestational diabetes, taking into account the synergistic effect of multiple SNP loci. We discovered that the T-G-T haplotypes significantly reduced the risk of developing gestational diabetes in the pre-BMI ≥ 24 group. Hence, the synergistic effect of multiple SNPs is a crucial aspect that requires further consideration and exploration.

This study has certain limitations that need to be acknowledged. Firstly, the sample size was not sufficient to allow for extensive association analysis. Additionally, the sample size for MG testing was even smaller due to the unavailability of serum samples from a larger number of recruits. Moreover, we were unable to measure *GLO1* activity as the previously described process was not easily implementable (33). Lastly, the study only included Chinese population subjects and did not explore other genetic backgrounds. Therefore, in the future, it is recommended that a larger sample size be collected to simultaneously test polymorphisms, *GLO1* enzyme activity, and MG levels to gain a better understanding of their relevance to GDM.

## 5 Conclusions

Based on these preliminary findings, it is possible that the *GLO1* genes, specifically rs4746 and rs1130534, may play a role in the susceptibility to GDM. Nevertheless, additional validation is essential to confirm this assertion.

## Data availability statement

The data presented in the study are deposited in the EVA repository, accession number are: Project: PRJEB64024 Analyses: ERZ19490372.

## Ethics statement

The studies involving humans were approved by Shunde Women and Children’s Hospital of Guangdong Medical University (Maternity and Child Healthcare Hospital of Shunde Foshan). The studies were conducted in accordance with the local legislation and institutional requirements. The participants provided their written informed consent to participate in this study.

## Author contributions

QZ, TY, and WW contributed equally to this study. QZ, WW, FH, and JYH collected clinical data and samples, QZ, TY, WW, and DZ did data analyzes, QZ, WW, TY, YW, and RG wrote the manuscript. JYH, JZH, and RG supervised the whole research. All authors contributed to the article and approved the submitted version.
